# Diagnosis of small pulmonary lesions by transbronchial lung biopsy with radial endobronchial ultrasound and virtual bronchoscopic navigation versus CT-guided transthoracic needle biopsy: A systematic review and meta-analysis

**DOI:** 10.1371/journal.pone.0191590

**Published:** 2018-01-22

**Authors:** Yeji Han, Hyun Jung Kim, Kyoung Ae Kong, Soo Jung Kim, Su Hwan Lee, Yon Ju Ryu, Jin Hwa Lee, Yookyoung Kim, Sung Shine Shim, Jung Hyun Chang

**Affiliations:** 1 Division of Pulmonary and Critical Care Medicine, Department of Internal Medicine, School of Medicine, Ewha Womans University, Seoul, Republic of Korea; 2 Department of Preventive Medicine, College of Medicine, Korea University, Seoul, Republic of Korea; 3 Department of Preventive Medicine, School of Medicine, Ewha Womans University, Seoul, Republic of Korea; 4 Department of Radiology, School of Medicine, Ewha Womans University, Seoul, Republic of Korea; Postgraduate Institute of Medical Education and Research, INDIA

## Abstract

**Background:**

Advances in bronchoscopy and CT-guided lung biopsy have improved the evaluation of small pulmonary lesions (PLs), leading to an increase in preoperative histological diagnosis.

We aimed to evaluate the efficacy and safety of transbronchial lung biopsy using radial endobronchial ultrasound and virtual bronchoscopic navigation (TBLB-rEBUS&VBN) and CT-guided transthoracic needle biopsy (CT-TNB) for tissue diagnosis of small PLs.

**Methods:**

A systematic search was performed in five electronic databases, including MEDLINE, EMBASE, Cochrane Library Central Register of Controlled Trials, Web of Science, and Scopus, for relevant studies in May 2016; the selected articles were assessed using meta-analysis. The articles were limited to those published after 2000 that studied small PLs ≤ 3 cm in diameter.

**Results:**

From 7345 records, 9 articles on the bronchoscopic (BR) approach and 15 articles on the percutaneous (PC) approach were selected. The pooled diagnostic yield was 75% (95% confidence interval [CI], 69–80) using the BR approach and 93% (95% CI, 90–96) using the PC approach. For PLs ≤ 2 cm, the PC approach (pooled diagnostic yield: 92%, 95% CI: 88–95) was superior to the BR approach (66%, 95% CI: 55–76). However, for PLs > 2 cm but ≤ 3 cm, the diagnostic yield using the BR approach was improved to 81% (95% CI, 75–85). Complications of pneumothorax and hemorrhage were rare with the BR approach but common with the PC approach.

**Conclusions:**

CT-TNB was superior to TBLB-rEBUS&VBN for the evaluation of small PLs. However, for lesions greater than 2 cm, the BR approach may be considered considering its diagnostic yield of over 80% and the low risk of procedure-related complications.

## Introduction

The increasing incidence of lung cancer is a worldwide issue. Exposure to tobacco smoke and environmental toxins is the main cause of an increase in the prevalence of lung cancer [1]. Small pulmonary lesions (PLs) may not be discernable in plain chest radiographs; however, the recent use of low-dose computer tomography (CT) for the screening of lung cancer has improved the PL detection rate [2]. A small, peripheral PL is usually defined as a solitary pulmonary lesion less than 3 cm in diameter and not visible endobronchially on routine flexible bronchoscopy [3]. The diagnosis of PLs less than 3 cm in diameter is a difficult problem, particularly when using bronchoscopic (BR) approaches. With the advent of new imaging modalities, such as multichannel CT and positron emission tomography, the demand for histological identification of small PLs has increased [2, 4, 5]. In addition, recent technological advances in bronchoscopy and CT guidance have improved tissue acquisition from small PLs [6–8] and reduced the need for invasive procedures, such as surgical lung biopsy, for lesions with radiological ambiguity [3].

Samples of small PLs can usually be acquired via the percutaneous (PC) or transbronchial route. Currently, the most accurate method through the PC route is CT-guided transthoracic needle biopsy (CT-TNB), an approach that evolved from fluoroscopy-guided TNB and has a diagnostic sensitivity of 82% to 99% [3]. However, the rate of complications, such as bleeding or pneumothorax, is higher using the PC route than using the BR approach [9]. Traditionally, the bronchoscopic approach for sampling small PLs has been fluoroscopy-guided transbronchial lung biopsy (TBLB). The advent of radial EBUS (rEBUS), which acquires tumor images by advancing a probe to the target lesion has further improved the yield of bronchoscopy [10]. In addition, using a guide sheath (GS) with rEBUS enhances diagnostic yield and shortens the procedure time [11]. A meta-analysis of rEBUS-TBLB demonstrated that the GS-assisted approach had a pooled sensitivity of 73% (95% confidence interval [CI], 70–76) [12]. Furthermore, virtual bronchoscopic navigation (VBN), a new method of image reconstruction using multidetector CT data, enables visualization of the path to the peripheral target lesion during bronchoscopy [7]. VBN combined with rEBUS-GS further improved the diagnostic yield to over 90% for lesions between 2 cm and 3 cm in diameter, a result that is comparable to the diagnostic yield of CT-TNB [13].

Several meta-analyses have specifically evaluated the diagnostic yields of the BR and PC approaches for small PLs [6, 12, 14–16]. Whether the BR or the PC should be the primary approach for the evaluation of small PLs remains controversial. Previous meta-analyses have been limited by differences in study design, heterogenous lesion size, procedure equipment and the approach. The diagnosis of small PLs can also be affected by the size of the lesion, availability of equipment and personal preference for certain methods. In particular, selecting non-surgical method of tissue acquisition should be determined by lesion size. When comparing the diagnostic yields of different methods, the lesions should be limited to those ≤ 3 cm in diameter [3, 13, 17].

The purpose of this meta-analysis was to compare the efficacy and safety of TBLB with rEBUS and VBN (TBLB-rEBUS&VBN), a BR approach, with CT-TNB, a PC approach, for tissue diagnosis of small PLs up to 3cm.

## Methods

### Literature search

A systematic literature search was performed in May 2016 for all studies describing biopsy of PLs using TBLB with rEBUS, GS and VBN or CT-TNB among five databases: MEDLINE, EMBASE, the Cochrane Library Central Register of Controlled Trials, Web of Science, and Scopus. Articles were identified using combinations of the following key words (http://dx.doi.org/10.17504/protocols.io.mfdc3i6 [PROTOCOL DOI]. The search terms were divided into four categories:

1. Lung lesion type: “lung,” “pulmonary,” “bronchial,” “neoplasms,” “cancer,” “lesion,” “tumor,” or “malignancy”;
2. Biopsy method: “biopsy,” “aspiration,” or “needle”;
3. Biopsy approach: “bronchoscopy,” “endobronchial ultrasound,” “radial EBUS,” “fluoroscopy,” “computed tomography,” or “CT-guided”; and
4. Additional techniques: “sheath” or “navigation.”

### Selection of studies and data extraction

**Fig 1:**
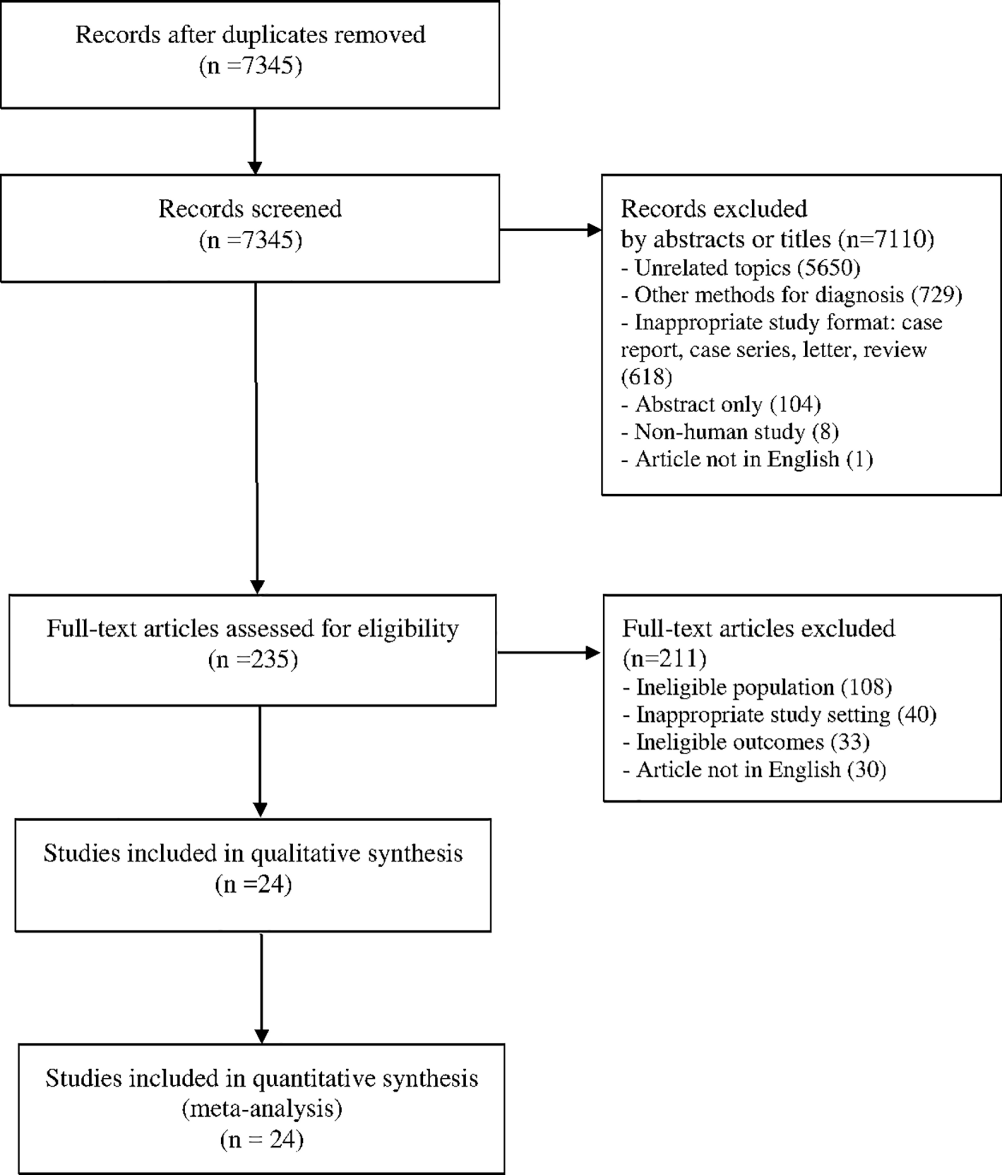
PRISMA flow diagram showing the study selection and data extraction processes.

We selected studies for the meta-analysis using the 2009 flow diagram provided by the PRISMA Group (Fig 1) [18]. The literature search was limited to papers published after 2000. The screening of each paper’s title and abstract was performed by one investigator (YJH). The full-text article was assessed for eligibility by two independent authors (YJH and SJK) based on the study inclusion criteria. In cases of disagreement, the decisions on paper selection were arbitrated by JHC.

Two authors (YJH and SJK) independently evaluated the characteristics of the selected papers and extracted data according to a standardized protocol (S1 Table). The following general characteristics of the studies were collected: author, countries in which the studies were conducted, year of publication, study design, number of subjects, procedure trial number, gender, age, definition of nodules, mean diameter, guidance methods, diagnostic yields, and the proportion of subjects with malignancy in the study sample. Data on the incidence of complications were also collected. For the criteria of diagnostic yield, the number of enrolled subjects and the number of procedures were recorded separately. Diagnostic yield was calculated using the following equation: diagnostic yield (%) = 100 x (number of correctly diagnosed cases / total number of biopsy procedures). The final retrieved data were reviewed by one author (JHC).

### Inclusion and exclusion criteria

The following inclusion criteria were used to select studies on TBLB-rEBUS&VBN:

1. rEBUS with a GS was used for the diagnosis of peripheral pulmonary lesions (PPLs).
2. Virtual bronchoscopy was used as a navigational method.
3. A PPL was defined as an endobronchial lesion not detected by bronchoscopy, and the size of these lesions was limited to ≤ 3 cm in diameter.
4. The final diagnosis was confirmed using the biopsy specimen or surgical specimen; in cases of diagnostic ambiguity, the final diagnosis was established at the clinical follow-up.

The following inclusion criteria were used to select studies on CT-TNB:

1. CT-guided transthoracic needle aspiration or biopsy, including conventional CT-guided transthoracic needle biopsy, CT fluoroscopy-guided transthoracic biopsy, and C-arm cone-beam CT-guided transthoracic biopsy, was used for the diagnosis of PL.
2. The biopsy target was a small PL ≤ 3 cm in diameter for which bronchoscopic biopsy was considered unfeasible based on the imaging information. The target lesion was described as a PPL, solitary pulmonary nodule (SPN), pulmonary nodule, ground glass opacity, or PL.
3. The final diagnosis was confirmed using the biopsy specimen or surgical specimen; in cases of diagnostic ambiguity, the final diagnosis was established at the clinical follow-up.

The following exclusion criteria were used when selecting studies on TBLB-rEBUS&VBN or CT-TNB:

1. Studies using non-human subjects, studies analyzing other methods of biopsy, studies written in a language other than English, or studies of an inappropriate type (case report, case series, letter, and review);
2. Central bronchial lesions evaluated by routine bronchoscopy;
3. Electromagnetic navigational bronchoscopy (ENB) procedure in TBLB;
4. Studies devoted to topics other than diagnostic outcomes with or without adverse events.

### Quality assessment

Two authors (YJH and JHC) independently examined the quality of the included studies using the Quality Assessment of Diagnostic Accuracy Studies 2 (QUADAS-2) tool [19]. We assessed the risk of bias and the concerns regarding applicability based on four parts of the assessment: patient selection, index test, reference standard, and flow and timing. Inconsistences were resolved by agreement between the authors (S2 Table).

### Statistical analysis

Meta-analyses were performed with Comprehensive Meta-Analysis software (version 3.0, Biostat, Englewood NJ, USA). The pooled diagnostic yields of TBLB-rEBUS&VBN and CT-TNB were calculated by the inverse-variance method with the logit-transformed diagnostic yields reported in each article. Pooled estimates of complications from CT-TNB were also extracted. Heterogeneity among study results was assessed by the I^2^ statistic, which describes the proportion of the variation across studies that is due to heterogeneity rather than chance. A value of I^2^ ≥ 50% was considered to indicate substantial heterogeneity. Heterogeneity was also evaluated with the conventional chi-squared test. When heterogeneity was considered low, the fixed effects model was performed; the random effects model was applied in cases of substantial heterogeneity. Forest plots were used to present the estimated diagnostic yield for each study and the overall pooled diagnostic yields. Publication bias was assessed using a funnel plot and Egger's linear regression test [20]. A P value < 0.05 was considered statistically significant in all analyses. To estimate the diagnostic yields by PL size, a subgroup analysis was performed on PLs ≤ 2 cm in diameter and PLs > 2 cm but ≤ 3 cm in diameter.

## Results

### Study selection and description

The database search identified 9285 papers. After excluding 1109 papers published before 2000 and 831 duplicates, 7345 papers remained. Based on the titles and abstracts, 235 papers were extracted for full-text review using the inclusion and exclusion criteria. Of these papers, 24 were finally included in the meta-analysis. The other 211 papers were excluded for the following reasons: 108 had an ineligible population, 40 had an inappropriate study setting, 33 had ineligible outcomes, and 30 were written in languages other than English (Fig 1).

The characteristics of the studies included in this analysis are shown in Table 1. The studies on TBLB-rEBUS&VBN and the studies on CT-TNB were analyzed separately. The former included 9 papers with a total of 813 procedures [13, 21–28]. All these studies were conducted in Japan between 2005 and 2016. Only one study was performed retrospectively. In 6 studies, the diagnostic yields were further analyzed by dividing lesions into two groups: ≤ 2 cm in diameter and 2–3 cm in diameter. The prevalence of malignancy ranged from 58% to 84%. A total of 15 studies utilized CT-TNB and comprised a total of 3463 procedures [8, 17, 29–41]. All included studies were conducted between 2000 and 2016. Only 2 studies were conducted in Western countries; the remaining studies were conducted in Far Eastern Asian countries. Five out of 15 studies used a prospective design (Table 1).

**Table 1:** Characteristics of the included studies in the TBLB-rEBUS&VBN and CT-TNB groups.

	Country	Year	Study design^1^	No. of subjects	No. of procedures^2^	No. of men (%)	Age (mean)	Patient selection	Mean diameter (mm)	Guidance method	Diagnostic yield (%)^3^, ≤ 3 cm	Diagnostic yield (%)^3^, ≤ 2 cm	Diagnostic yield (%)^3^, > 2 cm but ≤ 3 cm	Malignancy (%)^4^
**TBLB-rEBUS&VBN**														
Asahina H, *et al* [13]	Japan	2005	1	29	30	19 (66)	62	PPL ≤ 3 cm	18.9	Virtual Place (AZE)	19/30 (63)	8/18 (44)	11/12 (92)	77
Asano F, *et al*^6^ [21]	Japan	2008	1	24	24	UK	UK	PPL ≤ 3 cm	UK	Prototype (Olympus)	19/24 (79)	11/15 (73)	8/9 (89)	84
Ishida T, *et al*^6^ [22]	Japan	2011	1	102	99	64 (63)	69^5^	PPL ≤ 3 cm	18.0	Bf-NAVI (Cybernet)	80/99 (81)	44/58 (76)	36/41 (88)	80
Oshige M, *et al*^6^[23]	Japan	2011	1	40	40	UK	UK	PPL ≤ 3 cm	UK	Bf-NAVI (Olympus)	34/40 (85)	16/22 (73)	18/18 (100)	80
Tamiya M, *et al* [24]	Japan	2013	1	68	68	44 (65)	68^5^	PPL ≤ 3 cm	22.0	Lung-Point (Broncus)	53/68 (78)	20/27 (74)	33/41 (80)	63
Matsumoto Y, *et al* [25]	Japan	2015	2	121	121	68 (56)	UK	PPL ≤ 3 cm	UK	Ziostation2® (Ziosoft)	94/121 (78)	-	-	UK
Asano F, *et al*^6^ [26]	Japan	2015	1	99	99	UK	UK	PPL ≤ 3 cm	UK	Not mentioned	77/99 (78)	-	-	UK
Oki M, *et al* [27]	Japan	2015	1	305	305	178 (58%)	71^5^	PPL ≤ 3 cm	19.2^5^	Bf-NAVI (Cybernet)	203/305 (67)	92/162 (57)	111/143 (78)	58
Fukusumi M, *et al* [28]	Japan	2016	1	27	27	15 (56)	72^5^	PPL ≤ 3 cm	20.2	Ziostation2®, (Ziosoft)	17/27 (63)	-	-	67
**CT-TNB**														
Laurent F, *et al* [29]	France	2000	1	67	67	51 (76)	62	PN ≤ 2 cm	16.3	Conventional CT	-	61/67 (91)	-	71
Ohno Y, *et al* [30]	Japan	2003	1	162	162	97 (60)	67	SPN ≤ 2 cm	UK	Conventional CT	-	125/162 (77)	-	73
Yamagami T, *et al*^6^[31]	Japan	2003	1	102	102	UK	67	PN ≤ 3 cm	UK	CT fluoroscopy	92/102 (90)	61/68 (90)	31/34 (91)	UK
Yoshimatsu R, *et al*^6^[32]	Japan	2008	2	82	82	UK	UK	PN ≤ 3 cm	UK	CT fluoroscopy	79/82 (96)	62/63 (98)	17/19 (89)	UK
Hiraki T, *et al*^6^[17]	Japan	2009	2	795	795	UK	UK	PL ≤ 3 cm	UK	CT fluoroscopy	762/795 (96)	554/582 (95)	208/213 (98)	UK
Hwang HS, *et al* [33]	Korea	2010	2	27	27	18 (67)	67	PN ≤ 2 cm	12.0	CBCT	-	24/27 (89)	-	63
Inoue D, *et al*^6^ [34]	Japan	2012	2	65	65	UK	UK	GGO ≤ 2 cm	UK	CT fluoroscopy	-	62/65 (95)	-	UK
Choi MJ, *et al*^6^ [35]	Korea	2012	2	61	61	UK	UK	PL ≤ 3 cm	UK	CBCT	59/61 (97)	28/29 (97)	31/32 (97)	UK
Choi JW, *et al* [36]	Korea	2012	1	161	173	77 (48)	61	PN ≤ 2 cm	15.0	CBCT	-	160/173 (92)	-	54
Yamagami T, *et al* [37]	Japan	2013	2	73	85	35 (48)	69	GGO ≤ 3 cm	14.0	CT fluoroscopy	77/85 (91)	63/71 (89)	14/14 (100)	68
Lee SM, *et al*^6^ [38]	Korea	2014	2	758	758	UK	UK	PN ≤ 3 cm	UK	CBCT	734/758 (97)	468/485 (96)	266/273 (97)	66
Yang W, *et al* [39]	China	2015	1	311	311	211 (68)	60	SPN ≤ 3 cm	UK	Conventional CT	289/311 (93)	-	-	70
Takeshita J, *et al*^6^[40]	Japan	2015	2	391	391	UK	69	PPL ≤ 2 cm	UK	Conventional CT	-	344/391 (88)	-	UK
Jiao D, *et al* [41]	China	2016	2	60	60	27 (45)	52	PN ≤ 3 cm	23.4	CBCT	54/60 (90)	-	-	81
Rotolo N, *et al* [8]	Italy	2016	2	319	324	215 (67)	68	PN ≤ 3 cm	19.7	CBCT, CT fluoroscopy	279/324 (86)	-	-	64

Data are shown as a number, number (%), or case number/total number (%). Numbers in brackets denote references.

^1^Denoted by the following code: 1, prospective study design; and 2, retrospective study design.

^2^Defined as the number of lesions evaluated by the procedure.

^3^Calculated by the following equation: diagnostic yield (%) = 100 x (number of correctly diagnosed cases / total number of procedures).

^4^Defined as the proportion of patients who were confirmed to have malignant lesions among all subjects of a study.

^5^Data were presented as the median.

^6^Partly extracted from the number of total subjects.

Abbreviations: TBLB, transbronchial lung biopsy; rEBUS, radial endobronchial ultrasound; VBN, virtual bronchoscopic navigation; CT, computed tomography; TNB, transthoracic needle biopsy; PPL, peripheral pulmonary lesion; UK, unknown; SPN, solitary pulmonary nodule; GGO, ground glass opacity; PN, pulmonary nodule; PL, pulmonary lesion; and CBCT, cone-beam CT.

Evaluation with the QUADAS-2 tool revealed that the overall methodological quality was low or unclear due to potential bias regarding methodological quality and applicability. The choice between pathological confirmation by surgery or by clinical follow-up was not uniform and had a risk of bias. Most publications in the CT-TNB group used a retrospective study design, making it difficult to evaluate the consistency of patient selection (S2 Table). The funnel plots for PLs ≤ 3 cm showed general symmetry, and no publication bias was determined by Egger's linear regression test in the TBLB-rEBUS&VBN (P = 0.17, S1 Fig) and CT-TNB (P = 0.41, S2 Fig) groups.

### Outcome analysis

**Fig 2:**
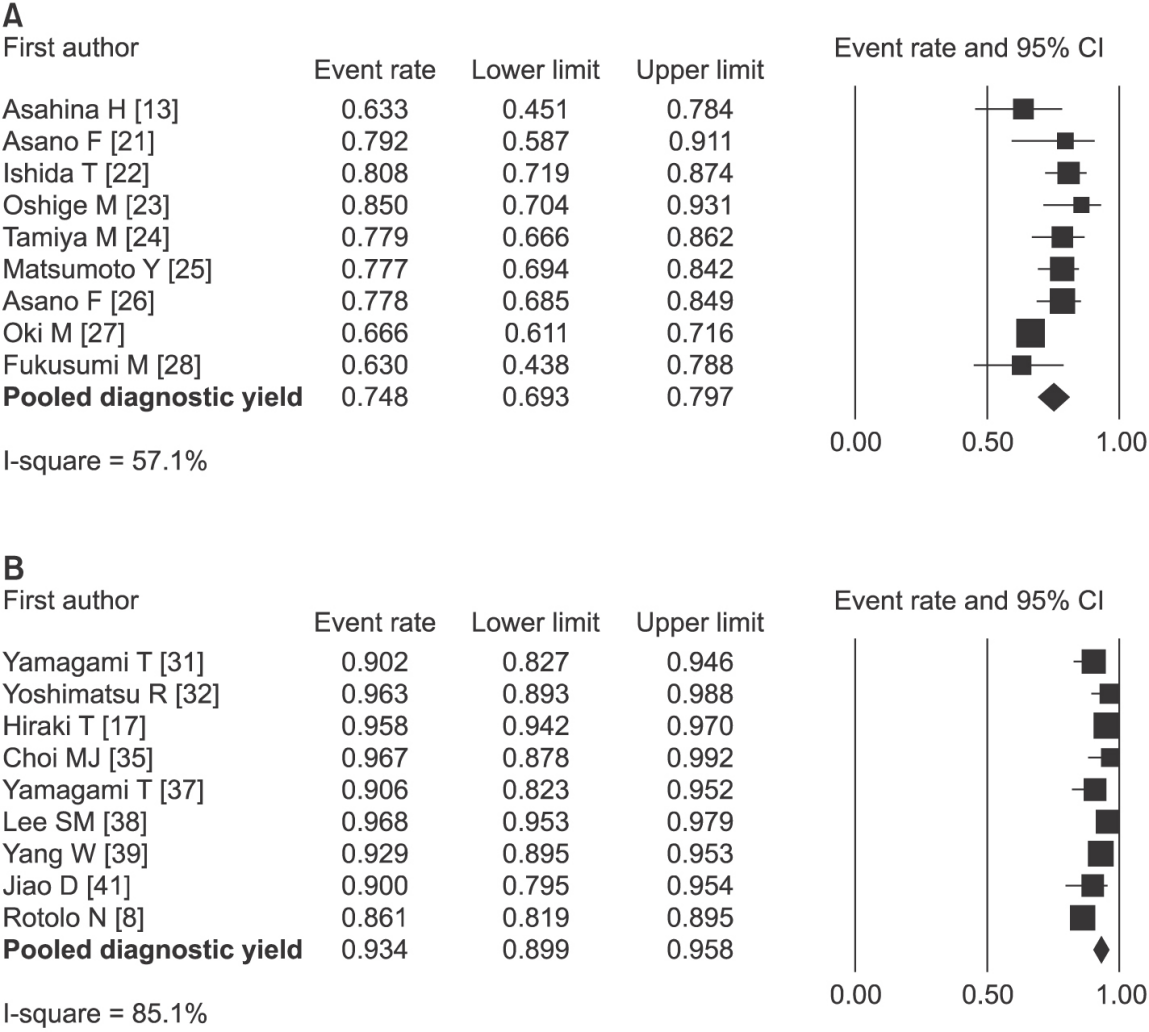
Forest plot of diagnostic yields for pulmonary lesions ≤ 3 cm. (A)TBLB-rEBUS&VBN. (B) CT-TNB.

For lesions with a diameter ≤ 3 cm, diagnostic yields in 813 procedures from 9 TBLB-rEBUS&VBN studies ranged from 63% to 85%. The pooled diagnostic yield was 75% (95% CI, 69–80) based on the forest plot. The I^2^ value was 57.1% (Fig 2A), indicating significant heterogeneity. Diagnostic yields in 2578 procedures from 9 CT-TNB studies ranged from 86% to 97%, with a pooled diagnostic yield of 93% (95% CI, 90–96). The I^2^ value was 85.1% (Fig 2B), indicating high heterogeneity.

**Fig 3:**
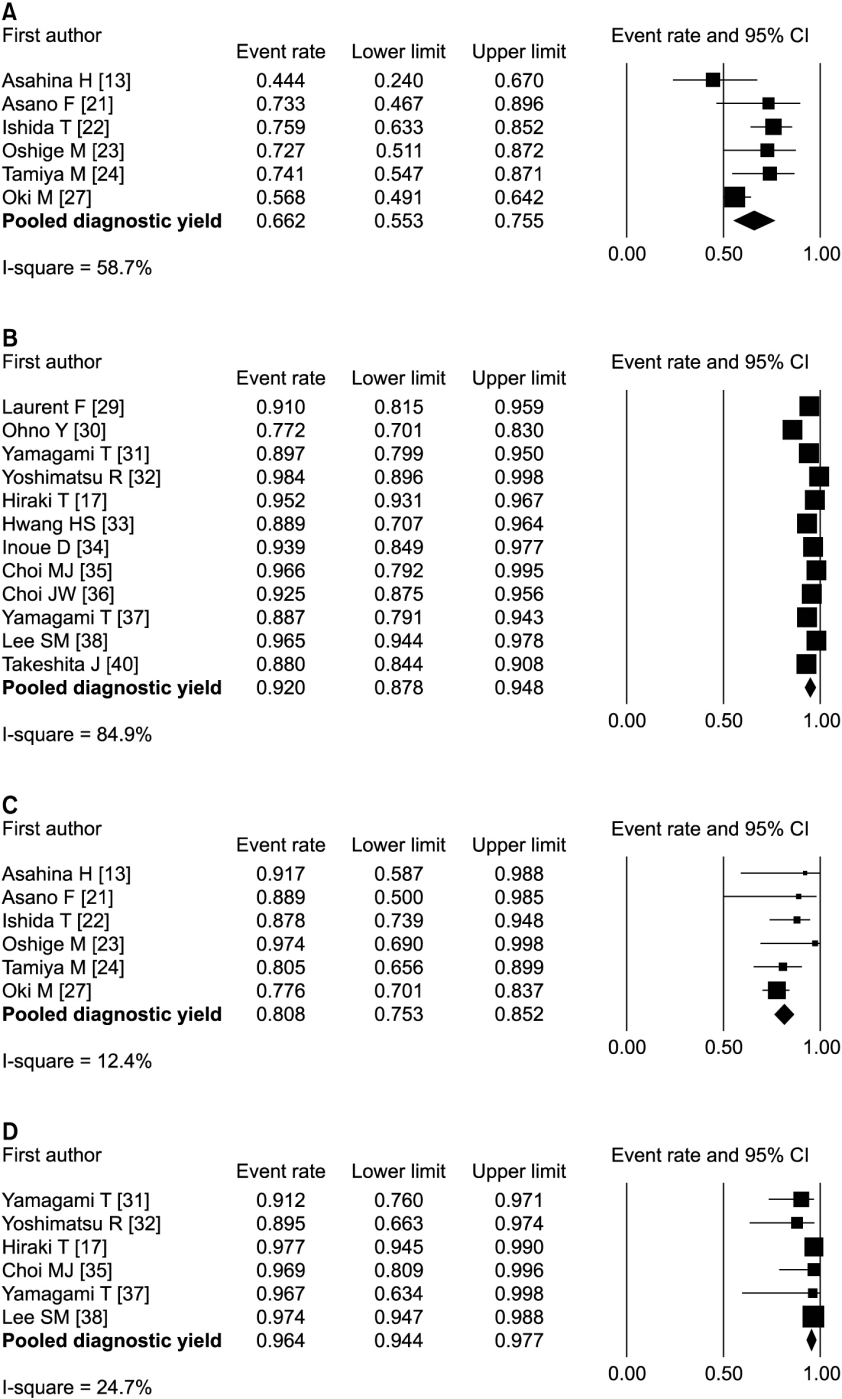
Forest plot of diagnostic yields for pulmonary lesions ≤ 2 cm and pulmonary lesions between 2 and 3 cm. (A) Lesions ≤ 2 cm in the TBLB-rEBUS&VBN group. (B) Lesions ≤ 2 cm in the CT-TNB group. (C) Lesions between 2 and 3 cm in the TBLB-rEBUS&VBN group. (D) Lesions between 2 and 3 cm in the CT-TNB group.

Subgroup analysis was performed on lesions ≤ 2 cm and lesions between 2 and 3 cm in diameter. In the TBLB-rEBUS&VBN group, the diagnostic yields for lesions ≤ 2 cm in 6 studies ranged from 44% to 76%, and the pooled diagnostic yield was 66% (95% CI, 55–76) (Fig 3A). The diagnostic yields for lesions between 2 and 3 cm in the same 6 studies were 78%–97%, and the pooled diagnostic yield was 81% (95% CI, 75–85) (Fig 3C). In the CT-TNB group, 12 studies with lesions ≤ 2 cm reported diagnostic yields of 77%–98% and a pooled diagnostic yield of 92% (95% CI, 88–95) (Fig 3B). Of these studies, 6 with lesions between 2 and 3 cm reported diagnostic yields of 90%–98% and a pooled diagnostic yield of 96% (95% CI, 94–98) (Fig 3D).

### Complications

Most of the studies included in the analysis reported complications associated with the procedure; however, 3 studies on TBLB-rEBUS&VBN and 2 studies on CT-TNB did not clearly report complications (Table 2). In the TBLB-rEBUS&VBN group, pneumothorax occurred in 10 subjects with a total of 426 procedures, and chest tube insertion and hemoptysis occurred in less than 1% of all procedures. In contrast, the rate of complications in the CT-TNB group (13 studies) was quite high. The incidence of pneumothorax as a post-procedural complication ranged from 15% to 52%, and the pooled complication rate was 26% (95% CI: 21–32) among 2611 procedures. Among these cases, the incidence of severe pneumothorax requiring chest tube insertion was 2%–8%, and the pooled incidence rate was 3% (95% CI: 1.8–4.8). Pulmonary hemorrhage was reported in 9 studies with an incidence rate ranging from 3% to 43%, and the pooled incidence rate was 16% (95% CI: 10–25) among 1545 procedures. Hemoptysis was reported in 8 studies with an incidence range of 2%-11%, and the pooled incidence rate was 7.1% (95% CI: 6.0–8.4) among 1865 procedures (Table 2).

**Table 2:** Complication rates of the included studies in the TBLB-rEBUS&VBN and CT-TNB groups.

	Pneumothorax	Chest tube^1^	Hemorrhage	Hemoptysis	Other
TBLB-rEBUS&VBN					
Asahina H, *et al* [13]	-	-	-	-	-
Asano F, *et al* [21]	0	-	-	0	-
Ishida T, *et al* [22]	0	-	-	0	-
Oshige M, *et al* [23]	0	-	-	0	-
Tamiya M, *et al* [24]	-	-	-	-	-
Matsumoto Y, *et al* [25]	2/121 (2%)	-	-	0	-
Asano F, *et al* [26]	-	-	-	-	-
Oki M, *et al* [27]	8/305 (3%)	3/305 (1%)	-	2/305 (1%)	2/305 (1%)^2^
Fukusumi M, *et al* [28]	0	0	-	0	-
CT-TNB					
Laurent F, *et al* [29]	10/67 (15%)	2/67 (3%)	29/67 (43%)	4/67 (6%)	-
Ohno Y, *et al* [30]	44/162 (27%)	3/162 (2%)	-	-	-
Yamagami T, *et al* [31]	38/110 (35%)	4/110 (4%)	32/110 (29%)	7/110 (6%)	1/110 (1%)^3^
Yoshimatsu R, *et al* [32] ^4^	28/82 (34%)	3/82 (3%)	23/82 (28%)	8/82 (10%)	-
Hiraki T, *et al* [17]	-	-	-	-	-
Hwang HS, *et al* [33]	5/27 (19%)	1/27 (3%)	1/27 (3%)	-	-
Inoue D, *et al* [34]	-	-	-	-	-
Choi MJ, *et al* [35] ^4^	10/61 (16%)	2/61 (3%)	-	1/61 (2%)	-
Choi JW, *et al* [36]	55/173 (32%)	3/173 (2%)	25/173 (14%)	-	2/173 (1%)^5^
Yamagami T, *et al* [37]	44/85 (52%)	3/85 (4%)	-	9/85 (11%)	-
Lee SM, *et al* [38]^4^	129/758 (17%)	9/758 (1%)	-	53/758 (7%)	-
Yang W, *et al* [39]	55/311 (18%)	3/311 (1%)	36/311 (12%)	11/311 (4%)	-
Takeshita J, *et al* [40] ^4^	144/391 (37%)	28/391 (7%)	14/391 (4%)	33/391 (8%)	8/391 (2%)^6^
Jiao D, *et al* [41]	9/60 (15%)	1/60 (2%)	5/60 (8%)	-	-
Rotolo N, *et al* [8]	89/324 (27%)	25/324 (8%)	65/324 (20%)	-	-
Pooled estimates (95% CI) ^7^	26%(21–32%)	3%(1.8–4.8%)	16%(10–25%)	7.1%(6.0–8.4%)	-

Data are shown as case number / total number (%). Numbers in brackets denote references.

^1^Occurred in cases of pneumothorax requiring chest tube insertion.

^2^Consisted of one case of pneumonia and one case of chest pain.

^3^Subcutaneous hematoma.

^4^Recalculated from the total subjects.

^5^Chest pain.

^6^Consisted of one or more of the following complications: air embolism, hypertension requiring treatment, posterior reversible encephalopathy syndrome, pain, shock, subcutaneous emphysema, subcutaneous hematoma, epilepsy, and bradycardia or tachycardia.

^7^Pooled from results related to CT-TNB.

Abbreviations: TBLB, transbronchial lung biopsy; rEBUS, radial endobronchial ultrasound; VBN, virtual bronchoscopic navigation; CT, computed tomography; TNB, transthoracic needle biopsy; and CI, confidence interval.

## Discussion

Since 2000, new methods of tissue acquisition that allow the early diagnosis of lung malignancy in small PLs have been devised and developed[7]. Nonetheless, the development of accurate and safe biopsy methods has always been challenging in patients with small PLs. Our study evaluated TBLB-rEBUS&VBN and CT-TNB, two methods with the highest diagnostic yields for small PLs, by systematic review and meta-analysis. For lesions of the same size, CT-guided lung biopsy exhibited a higher diagnostic yield than TBLB-rEBUS&VBN. For both techniques, diagnostic yield improved with increasing lesion size. For tissue biopsy of PLs < 2 cm, we recommend CT-TNB, which had a 26% better diagnostic yield than TBLB-rEBUS&VBN. For PLs 2–3 cm in diameter, we recommend TBLB-rEBUS&VBN after considering both the benefits and risks of the two methods. In detail, the 95% CI of the pooled diagnostic yield of TBLB-rEBUS&VBN for PLs 2–3 cm in diameter was 75%–85% and that of CT-TNB was 94%–98%. Interestingly, the risks associated with each biopsy method were the opposite: complications such as pneumothorax and hemoptysis were common with CT-TNB and rare with TBLB-rEBUS&VBN. Of note, the distribution of diagnostic yield was estimated to vary widely depending on the institution that conducted the study.

The results of previous studies on TBLB have shown different levels of success with different forms of image guidance. In cases using conventional fluoroscopy, diagnostic yields as low as 18%–62% were reported [15, 42]. A recent meta-analysis including several methods of guided bronchoscopy showed a pooled diagnostic yield of 70% (95% CI: 67–73) [6]. Radial EBUS was introduced in 1990 and have improved the diagnostic yield of biopsy [10]. The pooled diagnostic yield in a previous meta-analysis of rEBUS for PPLs of any diameter was 73% (95% CI: 70–76) [12], whereas the pooled diagnostic yield of rEBUS with VBN in our analysis was slightly better at 75% (95% CI: 69–80), even for a PL ≤ 3 cm. This suggests that the addition of VBN to rEBUS may have contributed to the improvement in diagnostic success. Another navigational method, ENB, is a newer technique that guides a bronchoscope to the pulmonary nodule in real time. Procedures using ENB were not included in the present analysis because difficulties were expected in evaluating various compounding variables. In a meta-analysis of ENB, the diagnostic yield was 65% (95% CI: 59–70) when used alone or in combination with other methods [14]. Another study reported that the combination of rEBUS and ENB increased the diagnostic yield from 69% to 88% [43]. If more precise endoscopic pathway information is obtained using adjunctive methods in TBLB, the diagnostic yield may approach that of CT-guided biopsy. Although not included in this study, the addition of rapid on-site evaluation (ROSE) is also known to significantly improve diagnostic yield. The addition of ROSE to TBLB using EBUS for PPLs ≤ 3 cm improved the diagnostic yield from 50% to 78% [44].

CT-guided lung biopsy has been the preferred method due to its simplicity and high diagnostic yield of > 90% [16]. Moreover, the use of CT enables three-dimensional planning, which minimizes the needle path, facilitates access to a central lesion or small nodule, and during the procedure, allows simultaneous imaging of the position of the needle and the location of a nodule [33]. A disadvantage of the transthoracic biopsy route for CT-guided lung biopsy is the higher incidence of complications, such as pneumothorax and pulmonary hemorrhage, than the transbronchial approach [9, 45–47]. Previous studies have reported a 15%–35% incidence of pneumothorax with this route [45, 46, 48, 49], and chest tube insertion was developed in 4.0%-6.6% of these cases [46, 49]. In our review, the incidence of pneumothorax was 26%, and the incidence of chest tube insertion due to pneumothorax was 3%, based on pooled estimates. The incidence of alveolar hemorrhage was also common, with severe hemoptysis in some cases [45]; hemorrhage has been reported to have an incidence rate of 2%–66% (median, 12%) in large-scale studies of CT-guided lung biopsy [46]. In our analysis, the pooled estimate of the incidence of alveolar hemorrhage was 16%, and that of the incidence of hemoptysis was 7.1%. Additionally, the incidence of pneumothorax has been reported to be increased when chronic obstructive pulmonary disease (COPD) is a comorbidity. The risk of pneumothorax is estimated to be approximately 54% in patients with emphysema, whereas it is as low as 15% in patients without emphysema [50]. There have been cases of very rare but fatal air embolism [51] and rare cases of tumor seeding along the needle tract [46, 48]. Because both optimal diagnostic yield and safety are difficult to achieve with either imaging-guided bronchoscopic biopsy or CT-guided lung biopsy, there are still debates about which method to choose. In reality, the selection of a biopsy method is greatly affected by the clinical environment, such as the facility, finances, and presence of an expert.

Previous meta-analyses have reported very different outcomes for each inclusion criterion. Heterogenous size criteria in previous studies have made it difficult to compare the outcomes from different methods. Direct comparison studies of image-guided TBLB with CT-TNB are rare [52–54]; however, one of these studies has shown an 87.5% diagnostic accuracy for rEBUS-TBLB, which was comparable to the 93.3% diagnostic accuracy of CT-guided lung biopsy [52]. The strength of our study is that all included studies were adjusted to the same size criteria and similar time periods, and analyzed separately based on two biopsy routes; moreover, the diagnostic yield and safety of those biopsy routes were compared.

However, this study also has several limitations. First, TBLB-rEBUS&VBN is not yet a universally utilized method, and studies that satisfied our inclusion criteria were all conducted in one country. It is possible that if the same studies were performed in different geographical regions, different results may be obtained. Second, the quality of the included studies in the TBLB-rEBUS&VBN and CT-TNB groups was not uniform. Third, the different imaging tools used in the CT-TNB group may lead to differences in the diagnostic yields. Fourth, our inclusion criteria may have bias in the selection of studies according to each biopsy route, and factors such as the anatomical location of the PL, distance from the pleura, nature of the PL, signs of the bronchus, and differences in the prevalence of malignancy may have influenced the diagnostic yields. The effects of these factors could not be properly corrected in this analysis. Fifth, randomized controlled trials that directly compared the two methods were rare; thus, a double-arm meta-analysis was not possible.

The two goals in tissue acquisition are to obtain a high diagnostic yield and to avoid procedure-related complications. Moreover, the primary biopsy route can be selected according to the preference and proficiency of each institution. Prior to selecting a primary tissue sampling method, clinicians should always consider the clinical and radiological information of a patient. In case of early lesions in which malignancy is suspected and surgery is feasible, TBLB-rEBUS&VBN may be recommended to prevent the rare possibility of tumor seeding along the needle tract during CT-guided lung biopsy [45, 52]. If the risk of percutaneous needle biopsy is predicted to be high, the bronchoscopic approach should be considered first. In the near future, as imaging techniques improve and minimally invasive methods further develop, the bronchoscopic approach for the diagnosis of a small PL may be a comparable alternative to CT-guided lung biopsy with enhanced diagnostic ability.

## Conclusions

The identification of an accurate and safe method for diagnosing small lung lesions has been a challenge. This meta-analysis of tissue acquisition methods for lesions 2–3 cm in diameter showed that TBLB combined with rEBUS and VBN achieves a relatively acceptable diagnostic yield with a lower risk of adverse events than CT-guided lung biopsy. For lesions ≤ 2 cm in diameter, CT-TNB is the best method among several current options. In the near future, technical improvements in image-guided bronchoscopy may further enhance diagnostic outcomes to a level closer to that of CT-guided biopsy.

## Supporting information

S1 Table PRISMA 2009 checklist. (DOC)

S2 Table Quality assessment of the included studies using the Quadas-2 tool. (PDF)

S1 Fig Funnel plot analysis of publication bias in the TBLB-rEBUS&VBN group. (TIF)

S2 Fig Funnel plot analysis of publication bias in the CT-TNB group. (TIF)

## Abbreviations

BR: bronchoscopic
CI: confidence interval
CT: computed tomography
CT-TNB: CT-guided transthoracic needle biopsy
GS: guide sheath
PC: percutaneous
PL: pulmonary lesion
PPL: peripheral pulmonary lesion
QUADAS-2: Quality Assessment of Diagnostic Accuracy Studies 2
rEBUS: radial endobronchial ultrasound
ROSE: rapid on-site evaluation
SPN: solitary pulmonary nodule
TBLB: transbronchial lung biopsy
VBN: virtual bronchoscopic navigation
