# Supplementary material for: Diagnosis of small pulmonary lesions by transbronchial lung biopsy with radial endobronchial ultrasound and virtual bronchoscopic navigation versus CT-guided transthoracic needle biopsy: A systematic review and meta-analysis
Source: PLoS One. 2018 Jan 22;13(1):e0191590. doi: 10.1371/journal.pone.0191590 (PMC5777651; doi:10.1371/journal.pone.0191590)
Supplement: S2 Table — (PDF) [file pone.0191590.s002.pdf]

S2 Table. Quality assessment of the included studies using the Quadas-2 tool

**Author**	**Risk of bias**				**Applicability concerns**		
	**Patient selection**	**Index test**	**Reference standard**	**Flow and timing**	**Patient selection**	**Index test**	**Reference standard**
TBLB-rEBUS&VBN							
Asahina H [13]	U	L	U	U	L	L	U
Asano F [21]	U	L	U	U	L	L	U
Ishida T [22]	U	L	L	U	L	L	L
Oshige M [23]	U	L	U	U	U	U	U
Tamiya M [24]	U	L	U	U	L	L	U
Matsumoto Y [25]	U	L	U	L	U	U	U
Asano F [26]	U	U	U	U	L	L	U
Oki M [27]	L	U	U	L	L	L	L
Fukusumi M [28]	U	L	U	U	L	U	U
CT-TNB							
Laurent F [29]	U	L	U	U	L	L	L
Ohno Y [30]	U	L	U	U	L	L	U
Yamagami T [31]	U	L	L	L	L	L	L
Yoshimatsu R [32]	U	L	U	U	U	L	U
Hiraki T [17]	U	U	U	L	L	L	L
Hwang HS [33]	U	L	L	U	L	L	U
Inoue D [34]	U	U	U	U	U	L	U
Choi MJ [35]	L	U	H	U	L	U	U
Choi JW [36]	U	L	U	U	L	L	U
Yamagami T [37]	U	U	U	L	L	U	L
Lee SM [38]	L	U	H	U	L	U	U
Yang W [39]	L	L	U	U	L	U	L
Takeshita J [40]	U	L	U	U	L	U	H
Jiao D [41]	U	U	U	U	L	U	U
Rotolo N [8]	L	L	H	U	L	L	U

H: high, L: low, U: unclear
