# Supplementary figures and images for: Diagnosis of small pulmonary lesions by transbronchial lung biopsy with radial endobronchial ultrasound and virtual bronchoscopic navigation versus CT-guided transthoracic needle biopsy: A systematic review and meta-analysis
Source: PLoS One. 2018 Jan 22;13(1):e0191590. doi: 10.1371/journal.pone.0191590 (PMC5777651; doi:10.1371/journal.pone.0191590)

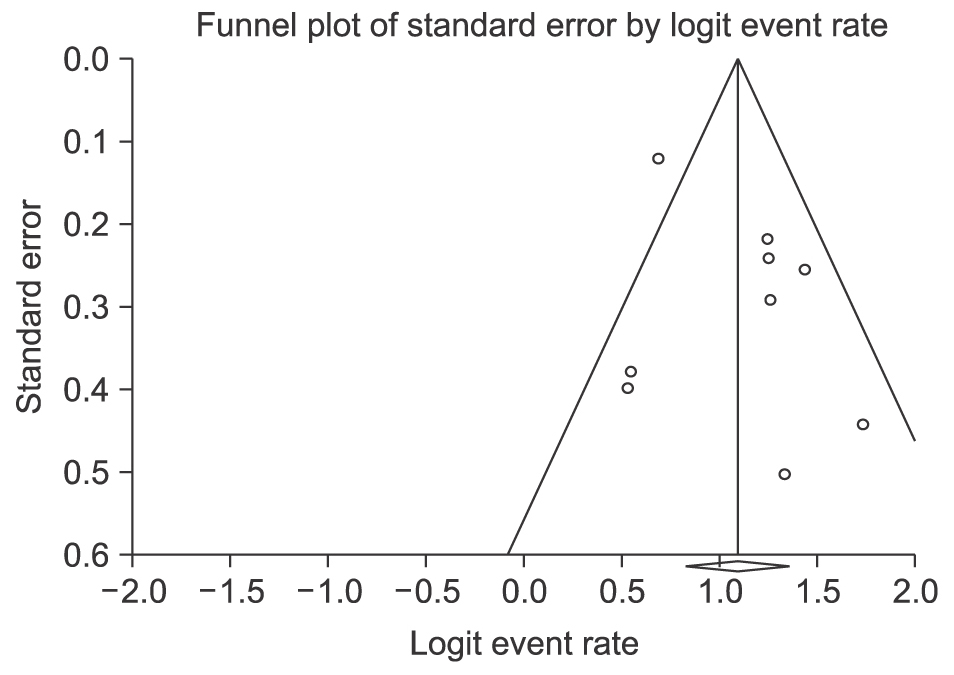

Supplement: S1 Fig — (TIF) [file pone.0191590.s003.tif]

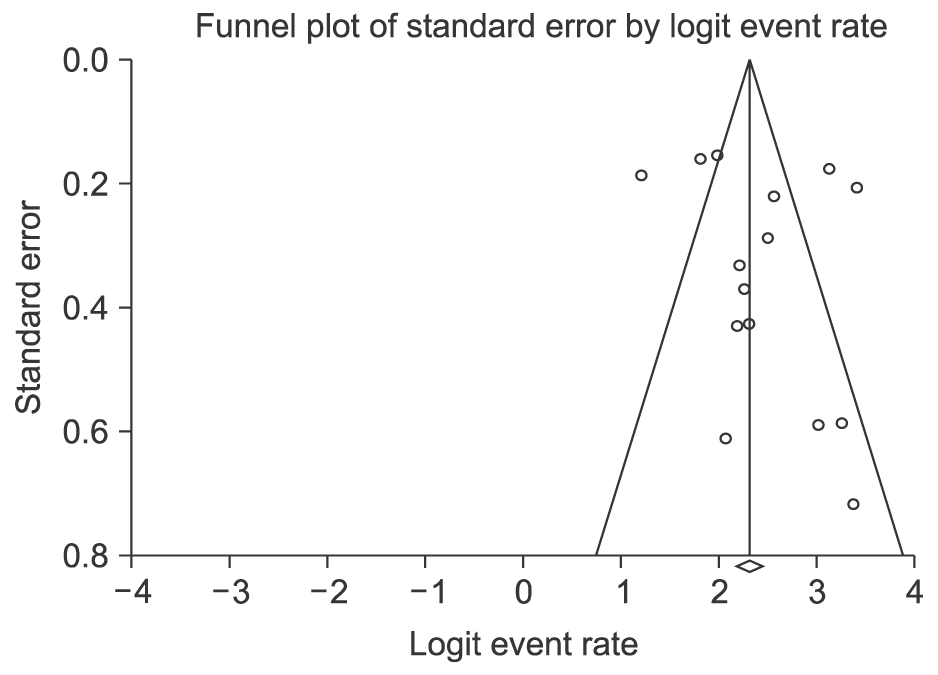

Supplement: S2 Fig — (TIF) [file pone.0191590.s004.tif]
